# Quercetin Regulates Autophagy to Inhibit PRRSV Replication Through the PI3K/Akt/mTOR Signaling Pathway

**DOI:** 10.3390/v17121637

**Published:** 2025-12-17

**Authors:** Yuxin Yang, Xinmiao Li, Haitao Shi, Jiaying Yu, Chen Gao, Yuanhong Liu, Wenjun Feng, Luyuan Peng, Bendong Fu, Pengfei Yi

**Affiliations:** 1 College of Veterinary Medicine, Jilin University, Changchun 130062, China; a1454341871@foxmail.com (Y.Y.); xinmiao22@mails.jlu.edu.cn (X.L.);; 2 Experiment Management Center, Dezhou University, Dezhou 253023, China

**Keywords:** PRRSV, quercetin, autophagy

## Abstract

Porcine Reproductive and Respiratory Syndrome (PRRS), caused by the Porcine Reproductive and Respiratory Syndrome Virus (PRRSV), is a highly contagious viral disease responsible for significant economic losses in the global swine industry. Quercetin, a polyphenolic flavonoid known for its antiviral properties, was investigated in this study for its ability to inhibit PRRSV replication by modulating autophagy. Our study demonstrates that quercetin can inhibit PRRSV replication in MARC-45 cells by regulating the degradation of autophagosomes and suppressing the generation of autophagosome. We further suggest that quercetin inhibits PRRSV-induced autophagy via the PI3K/Akt/mTOR signaling pathway, suppressing autophagosome formation while promoting autophagosome-lysosome fusion, ultimately leading to reduced PRRSV replication. In conclusion, our study demonstrates that quercetin inhibits PRRSV replication by regulating autophagy through the PI3K/Akt/mTOR pathway.

## 1. Introduction

Porcine Reproductive and Respiratory Syndrome (PRRS), first identified in 1987 in the United States, is one of the most economically significant diseases affecting the global swine industry [1]. PRRS is an acute, highly contagious disease caused by Porcine Reproductive and Respiratory Syndrome Virus (PRRSV) [2]. It primarily affects weaned piglets and breeding sows. The infection is characterized by reproductive failures, such as abortions and stillbirths, in sows, and severe respiratory symptoms in piglets [3]. PRRSV is an enveloped, single-stranded, positive-sense RNA virus belonging to the genus *Porartevirus*, the family *Arteriviridae*, and the order *Nidovirales*. Due to similarities in genome organization and expression strategies, arteriviruses and coronaviruses are classified within the order *Nidovirales* [4]. The PRRSV genome consists of a single-stranded, positive-sense RNA molecule approximately 15 kb in length, featuring a 5′-cap and a 3′-polyadenylation tail [5]. It encodes 11 open reading frames (ORFs): ORF1a, ORF1b, ORF2a, ORF2b, ORFs 3 to 7, as well as the newly identified ORF5a and ORF2 (TF). ORF1a and ORF1b comprise approximately 75% of the genome and partially overlap, while the remaining 25% encodes eight structural proteins: seven membrane proteins (GP2a, GP2b[E], GP3, GP4, GP5a, GP5, and M) and the nucleocapsid protein (N) [6,7].

Autophagy, a conserved catabolic process involved in the degradation of proteins and organelles to maintain cellular homeostasis [8], plays a critical role in PRRS [9]. PRRSV hijacks the host cell’s autophagy machinery [10], initiating autophagy through Ca^2+^ signaling and activation of autophagy-related proteins such as UNC-51-like kinase 1(ULK1) [11,12,13]. PRRSV induces the formation of autophagosomes, double-membrane vesicles (DMVs) that provide sites for its replication. When the autophagosome fuses with the lysosome to form an autolysosome, the viral material it contains is delivered for degradation [9,14]. To evade autophagic degradation, however, PRRSV employs strategies to prevent the fusion of autophagosomes with lysosomes [10,15]. Additionally, PRRSV can degrade host factors via autophagy to evade immune responses [16,17]. While the precise mechanisms linking PRRSV infection to autophagy remain to be fully elucidated, it is clear that regulating autophagy can inhibit PRRSV replication.

Quercetin, a bioactive flavonoid, is ubiquitously present in numerous edible and medicinal plants. Among flavonoids, quercetin has attracted considerable scientific interest for its strong antiviral properties [18,19]. For instance, quercetin has demonstrated a dose-dependent inhibitory effect on Zika virus [20] replication. Furthermore, many herbal formulations with antiviral properties include quercetin as a principal active component [21,22]. For example, the Hua-Shi-Bai-Du decoction exhibits anti-SARS-CoV-2 activity, with quercetin and its derivatives accounting for 1.838% of its active constituents [23]. Studies suggest that quercetin regulates lysosome-dependent autophagy, contributing to the attenuation of disease progression [24]. These observations prompted us to explore whether quercetin can inhibit PRRSV infection by modulating autophagy.

## 2. Materials and Methods

### 2.1. Reagents, Cells, and Virus

Quercetin (>99% purity) was sourced from Chengdu Must Bio-Technology Co., Ltd. (Chengdu, China). Rapamycin (HY-10219) and MHY1485 (HY-B0795) were sourced from MCE (Shanghai, China). Chloroquine (CQ) (C6628) was sourced from Sigma-Aldrich (St. Louis, MO, USA). Akt antibody (HY-P80535), p-Akt antibody (HY-P80276), PI3K antibody (HY-P80867), p-PI3K antibody (HY-P80846), p-mTOR antibody (HY-P80469), p62 antibody (HY-P80899), and Beclin-1 antibody (HY-P80568) were sourced from MCE (Shanghai, China). LC3 antibody (T55992), ATG antibody (5T55766), and ULK1 antibody (T56902) were sourced from Abmart (Shanghai, China). mTOR Monoclonal antibody and β-actin Monoclonal antibody (66009-1-Ig) were sourced from Proteintech (Wuhan, China). Peroxidase-conjugated Affinipure Goat Anti-Mouse IgG (H+L) (SA00001-1) and HRP-conjugated Affinipure Goat Anti-Rabbit IgG (H+L) (SA00001-2) were sourced from Proteintech (Wuhan, China).

Marc-145 cells (African green monkey kidney epithelial cell line, ATCC, CRL-12219.) were cultured in Dulbecco’s modified Eagle’s medium-high glucose (Sigma-aldrich, St. Louis, MO, USA) containing 10% fetal bovine serum (FBS; Gibco, Thermo Fisher Scientific, Waltham, MA, USA) at 37 °C under 5% CO_2_.

The PRRSV-JL/07/SW strain was gifted by the Laboratory of Animal Infectious Diseases, College of Animal Medicine, Jilin University. The viral titer was 10^6.25^TCID_50_/0.1 mL via the endpoint dilution method.

### 2.2. Cell Viability Analysis (CCK-8)

MARC-145 cells were cultured in 96-well plates at a density of 5 × 10^3^ cells/well and incubated at 37 °C for 24 h. Then, the cells were treated with quercetin at different concentrations (0–100 μg/mL) and continued to be cultured at 37 °C under 5% CO_2_ for 48 h. Subsequently, cell viability was measured using the CCK8 kit (Absin, Shanghai, China) according to the manufacturer’s guidelines.

### 2.3. In Vitro Infection with PRRSV

MARC-145 cells were cultured in 6-well plates at a density of 1 × 10^5^ cells/well and incubated in an incubator at 37 °C under 5% CO_2_ for 24 h. Cells were then infected with PRRSV (MOI of 0.8) for 2 h at 4 °C. 1 mL/well of PBS was used to wash the cells 2–3 times to remove unadsorbed cells. Cells were cultured for 48 h with the addition of maintenance solution containing 2% FBS per well.

### 2.4. Treatment with Drugs and Reagents

Quercetin was prepared as 12.5 μg/mL, 6.25 μg/mL, and 3.125 μg/mL solutions using medium with 2% FBS. Rapamycin (Rapa) and MHY1485 were dissolved in DMSO at a solubility of 100 nM and 10 μM. CQ was dissolved in PBS at a concentration of 25 μM. Following a 2 h incubation with PRRSV, the DMEM-high glucose was removed, and 2 mL solutions of Rapa, MHY1485 and CQ were added to the corresponding wells.

### 2.5. Quantitative Reverse Transcription Polymerase Chain Reaction (RT-qPCR)

Total RNA was extracted using the RNeasy Mini Kit (Qiagen, Hilden, Germany) according to the manufacturer’s instructions. mRNA was reverse transcribed into cDNA using the Reverse Transcription Kit (TRAN, Beijing, China). Relevant gene expression was measured using SYBR Premix Ex Taq™ (Takara, Shiga, Japan). Relative expression levels were determined using the 2^−∆∆Ct^ method with GAPDH mRNA as a reference. For details of the relevant primers, please refer to Table 1.

### 2.6. Western Blot

Total protein was extracted using RIPA buffer (Thermo Fisher, USA) containing a mixture of phosphatase and protease inhibitors. Total protein concentration was determined using the BCA kit (Thermo Fisher, USA). Equal amounts of protein were separated by SDS-PAGE. The separated proteins were transferred to a PVDF membrane (Millipore, Boston, MA, USA). The proteins were subsequently conjugated with specific antibodies and detected. Grayscale analysis was performed using ImageJ (Fiji v2.15.0).

### 2.7. Transmission Electron Microscope (TEM)

Forty-eight hours after PRRSV infection of MARC-145 cells, the cells were collected and then fixed with 2.5% glutaraldehyde for 12 h at 4 °C, post-fixed in 1% osmium tetroxide, dehydrated in gradient ethanol, and embedded in epoxy resin. Next, ultrathin sections were stained with uranyl acetate and lead citrate. Finally, autophagosome-like vesicles were observed using TEM (Ht7800/Ht7700; Hitachi, Tokyo, Japan).

### 2.8. Monodansylcadaverine Staining (MDC)

For MDC, 1 × 10^5^ cells were cultured in 6-well plates overnight according to the manufacturer’s instructions. After different treatments, cells were stained at 37 °C for 30 min in the dark, washed 3 times with assay buffer and visualized using a fluorescence microscope (Olympus, Tokyo, Japan).

### 2.9. Immunofluorescence Analysis (IFA)

Cells seeded on a 12-well glass slide were washed three times with PBS and then fixed with 4% paraformaldehyde. Cells were then washed three times with PBS and then permeabilized with 0.2% Triton X-100 for 10 min. Subsequently, cells were washed three times with PBS and occluded for 1 h using 5% Albumin Bovine Ⅴ (A6020, Biotopped, Beijing, China). This was followed by overnight incubation with specific primary antibodies. Cells were washed three times with PBS to remove excess antibody, incubated with specific secondary antibody for 1 h, washed three times with PBS to remove excess antibody, and restained using DAPI for 10 min. Cells were imaged using a fluorescence microscope.

### 2.10. Statistical Analysis

All data were expressed as means ± standard deviation (SD). Analysis of variance (ANOVA) was performed and plotted using GraphPad Prism 6.0 software. *p* < 0.05 was considered statistically significant.

## 3. Results

### 3.1. Quercetin Inhibits PRRSV Replication

**Figure 1:**
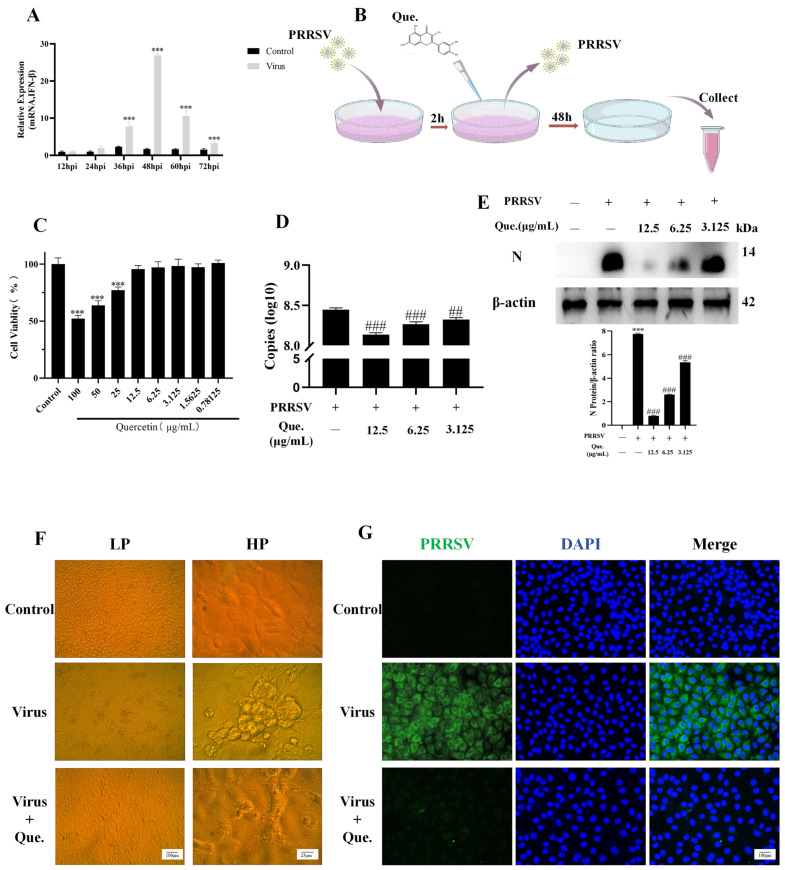
Quercetin inhibits viral replication. (**A**) IFN-β mRNA expression in PRRSV-infected MARC-145 cells at indicated time points (12–72 h). (**B**) Procession of PRRSV infection and quercetin treatment. (**C**) Treatment of uninfected MARC-145 cells with increasing concentrations of quercetin for 48 h. (**D**) ORF7 mRNA expression of PRRSV (MOI of 0.8) at different concentrations of quercetin. (**E**) PRRSV N protein expression. (**F**) MARC-145 cells were infected with PRRSV (MOI of 0.8) and treated with quercetin (12.5 μg/mL) for 48 hpi. Cytopathy was observed by optical microscope (LP, low power field. HP, High power field). Scale bar, 100 μm/25 μm. (**G**) MARC-145 cells were infected with PRRSV (MOI of 0.8) and treated with quercetin (12.5 μg/mL) for 48 hpi. Fluorescence intensity of N protein was observed by fluorescence microscopy. Scale bar, 100 μm. *** *p* < 0.001, compared with control. ## *p* < 0.01; ### *p* < 0.001, compared to the viral group.

First, we infected MARC-145 cells with PRRSV and detected the mRNA expression of IFN. At 48 h post-infection (hpi), the most statistically significant difference in IFN-β expression was observed between the control group and the virus-infected group, so we selected 48 hpi for the subsequent experiments. PRRSV infected MARC-145 cells for 2 h. Then, the cells were washed twice with PBS and treated with quercetin. After 48 h, samples were collected (Figure 1B). To determine the safe concentration of quercetin for MARC-145 cells, we assessed the survival rate of MARC-145 cells after incubation with varying concentrations of quercetin. As shown in Figure 1C, quercetin concentrations of 12.5 μg/mL and below had no significant effect on cell survival. Therefore, we chose the concentrations of 12.5 μg/mL, 6.25 μg/mL and 3.125 μg/mL for the subsequent experiments. Quercetin could inhibit the expression of ORF7 mRNA (Figure 1D) and N protein (Figure 1E). Cytopathic effects could be seen when PRRSV was persistently infected for 48 h, and quercetin could reverse this phenomenon (Figure 1F). Immunofluorescence (IF) staining results showed that quercetin could reduce the fluorescence intensity of viral N protein (Figure 1G).

### 3.2. Quercetin Inhibits PRRSV-Induced Autophagy

**Figure 2:**
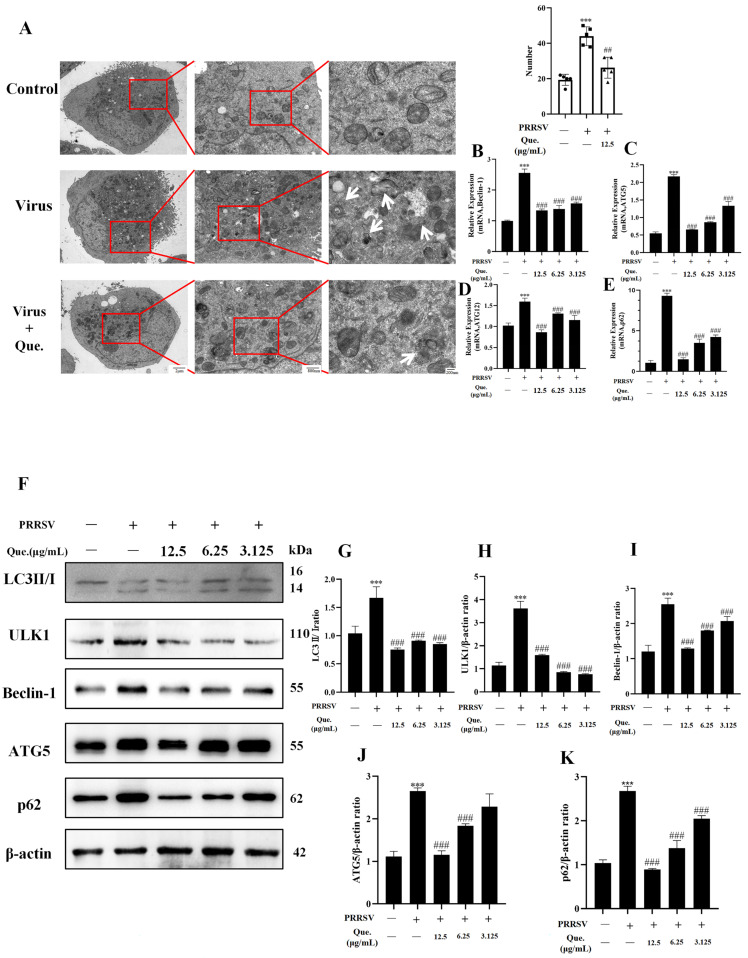
Quercetin inhibits PRRSV-induced autophagy. (**A**) Observation of 48 hpi quercetin (12.5 μg/mL) treatment with or without the number of autophagosome in MARC-145 cells by TEM. White arrows point to autophagosomes (MOI of 0.8). (**B**–**E**) MARC-145 cells were infected with PRRSV (MOI of 0.8) and treated with quercetin for 48 h. Beclin-1 mRNA (**B**), p62 mRNA (**C**), ATG5 mRNA (**D**) and ATG12 mRNA (**E**). (**F**–**K**) MARC-145 cells were infected with PRRSV and treated with quercetin for 48 h. LC3II/I (**G**), ULK1 (**H**), Beclin-1 (**I**), ATG5 (**J**) and p62 (**K**). *** *p* < 0.001, compared with control. ## *p* < 0.01; ### *p* < 0.001, compared to virus.

**Figure 3:**
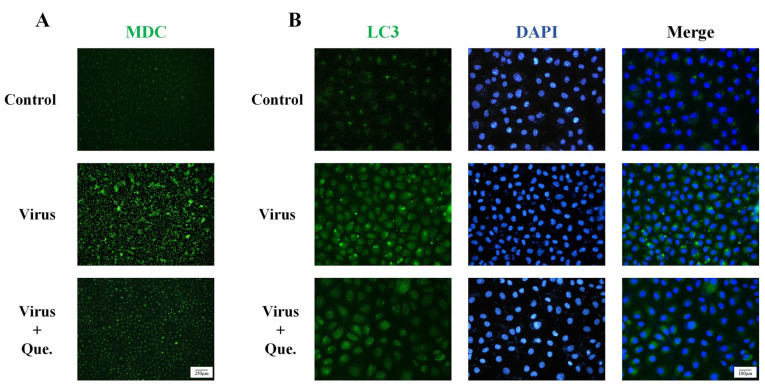
Quercetin decreases autophagy in MARC-145 cells. (**A**) MARC-145 cells were infected with PRRSV (MOI of 0.8) and treated with quercetin (12.5 μg/mL) for 48 h. Autophagosomes were detected with MDC. Scale bar, 250 μm. (**B**) Detection of LC3 protein immunofluorescence intensity by fluorescence microscopy. Scale bar, 100 μm.

Previous studies have demonstrated that PRRSV infection of MARC-145 cells in vitro leads to autophagy Our findings indicate that quercetin treatment reversed this effect. We used two autophagy modulators that act independently of the PRRSV: Rapa, which promotes autophagosome formation, and CQ, which inhibits autophagosome degradation. Both induce autophagosome accumulation. However, when cells were co-treated with quercetin and either Rapa or CQ, autophagosome levels were reduced (Figure S1). Transmission electron microscopy (Figure 2A) revealed that PRRSV infection resulted in the accumulation of autophagosomes within the cells, which was subsequently reversed by quercetin. In MARC-145 cells under persistent PRRSV infection for 48 h, the mRNA expression of autophagy-related genes—Beclin-1, p62, ATG5, and ATG12—was significantly elevated. Notably, quercetin led to a reduction in the expression levels of these autophagy-related genes (Figure 2B–E). Western blot analysis indicated that the expression of LC3 II/I, ULK1, Beclin-1, ATG5 and p62 was significantly lower in the quercetin group compared to the viral group (Figure 2F–K). Additionally, results from MDC (Figure 3A) and IF (Figure 3B) confirmed that autophagy was diminished following quercetin treatment. These results clearly demonstrate that quercetin can inhibit autophagy promoted by PRRSV.

### 3.3. Quercetin Inhibits PRRSV Replication by Suppressing Autophagy

**Figure 4:**
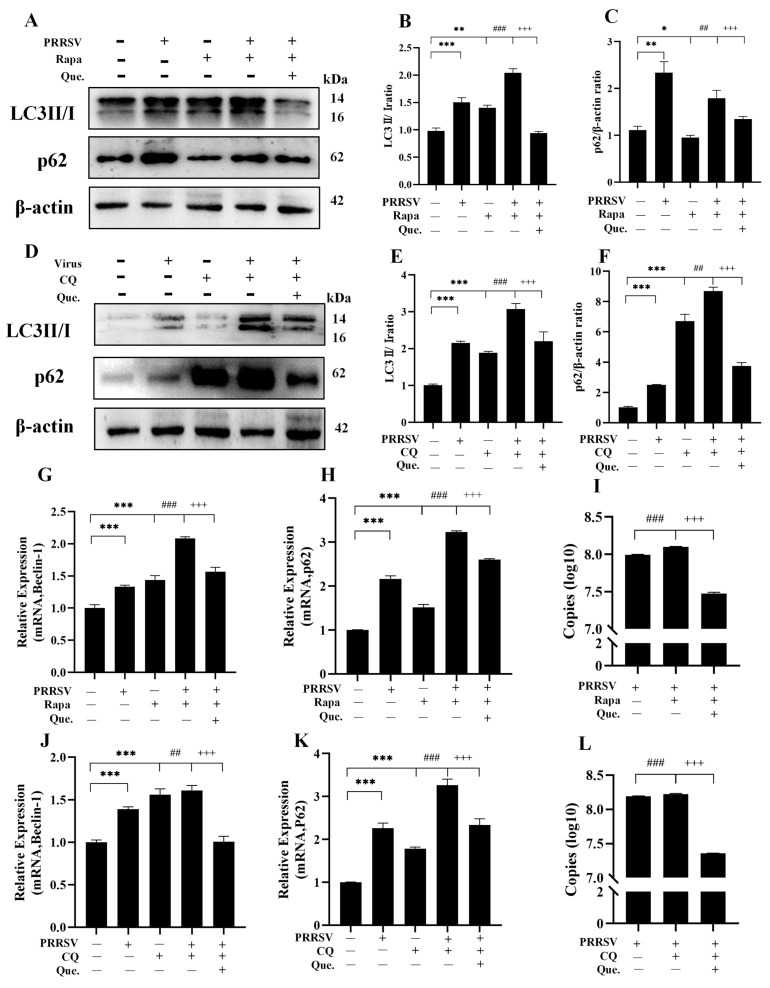
Quercetin inhibits PRRSV replication by suppressing autophagy. (**A**–**C**) MARC-145 cells infected with PRRSV (MOI of 0.8) infected for 48 h, MARC-145 cells treated with quercetin (12.5 μg/mL) and Rapa (100 nM), LC3II/I (**B**) and p62 (**C**). (**D**–**F**) PRRSV-infected MARC-145 cells, 48 h. Quercetin (12.5 μg/mL)- and CQ (25 μM)-treated MARC-145 cells, LC3II/Ⅰ (**E**) and p62 (**F**). (**G**–**I**) PRRSV (MOI of 0.8)-infected MARC-145 cells, 48 h. Quercetin (12.5 μg/mL)- and Rapa (100 nM) = treated MARC-145 cells, Beclin-1 mRNA (**G**), p62 mRNA (**H**) and ORF7 mRNA (**I**). (**J**–**L**) PRRSV-infected MARC-145 cells, 48 h. Quercetin (12.5 μg/mL)- and CQ (25 μM)-treated MARC-145 cells, Beclin-1 mRNA (**J**), p62 mRNA (**K**) and ORF7 mRNA (**L**). * *p* < 0.05; ** *p* < 0.01; *** *p* < 0.001, compared with control. ## *p* < 0.01; ### *p* < 0.001, compared to the viral group. +++ *p* < 0.001, compared to the Rapa/CQ group.

To investigate whether quercetin inhibits PRRSV infection via the autophagy pathway, we performed further experiments using the autophagy activator Rapa and the inhibitor CQ. Rapa induces autophagy flux by inhibiting mTOR. The addition of Rapa partially restored the autophagy flux that was suppressed by viral infection. Compared to the group of infected cells treated with Rapa alone, the group treated with both Rapa and quercetin showed a decreased ratio of LC3II/I (Figure 4B), the expression of Beclin-1 mRNA (Figure 4G) reduced, reduced p62 protein (Figure 4C) and mRNA (Figure 4H) levels, diminished autophagosome formation, and significantly decreased viral copy numbers (Figure 4I), suggesting that quercetin inhibits PRRSV replication by suppressing autophagosome generation. CQ blocks autophagosome degradation. Compared to the group of infected cells treated with CQ alone, the group treated with both CQ and quercetin showed a decreased LC3-II/I ratio (Figure 4E), reduced p62 protein levels (Figure 4F), and downregulated mRNA expression of Beclin-1 (Figure 4J) and p62 (Figure 4K), indicating that the blockade of autophagosome degradation was alleviated, alongside a decrease in viral load (Figure 4L). When autophagic flux was blocked by CQ during quercetin treatment of PRRSV infection, the accumulation of autophagosomes decreased (Figure S2A). This reduction attenuated the inhibitory effect of quercetin on viral replication (Figure S2B). These results demonstrate that quercetin also inhibits PRRSV replication by promoting autophagosome degradation. In summary, quercetin suppresses PRRSV replication through dual regulation of the autophagy pathway.

### 3.4. Quercetin Suppresses the PI3K/Akt/mTOR Signaling Pathway

**Figure 5:**
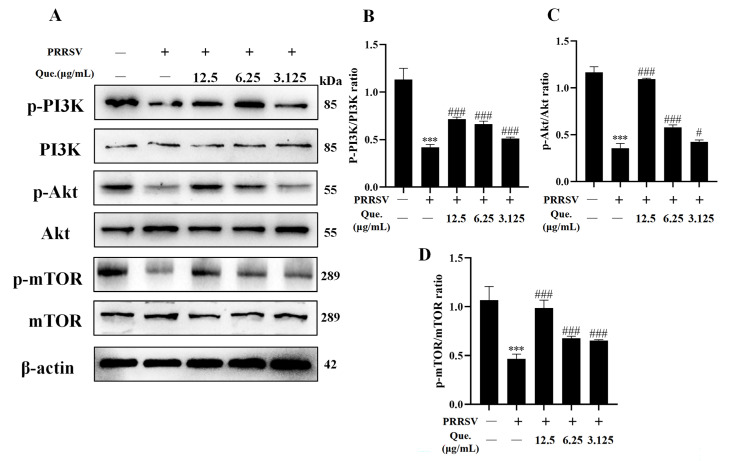
Quercetin suppresses the PI3K/Akt/mTOR signaling pathway. (**A**–**D**) MARC-145 cells were infected with PRRSV (MOI of 0.8) and treated with quercetin for 48 h. p-PI3K/PI3K ratio (**B**), p-Akt/Akt (**C**), p-mTOR/mTOR (**D**). *** *p* < 0.001, compared to control. # *p* < 0.05; ### *p* < 0.001, compared to the viral group.

The PI3K/Akt/mTOR pathway serves as a crucial regulator of autophagy, and PRRSV infection has been shown to modulate autophagy through this signaling axis. As illustrated in Figure 5, at 48 h post-PRRSV infection, the phosphorylation levels of PI3K, Akt, and mTOR were significantly decreased. Treatment of infected cells with quercetin restored the phosphorylation of PI3K, Akt, and mTOR. Collectively, these results suggest that quercetin inhibits PRRSV replication and concurrently counteracts the virus-induced suppression of the PI3K/Akt/mTOR pathway.

### 3.5. Quercetin Inhibits PRRSV Replication via the PI3K/Akt/mTOR Signaling Pathway

**Figure 6:**
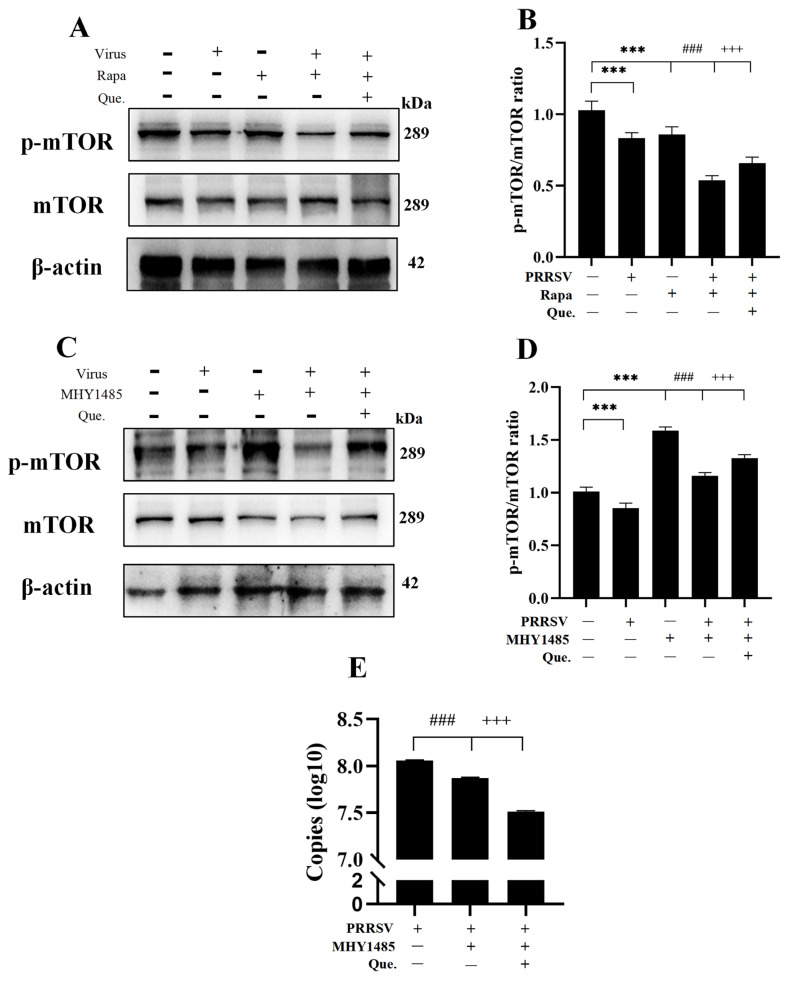
Quercetin inhibits PRRSV replication via the PI3K/Akt/mTOR signaling pathway. (**A**,**B**) PRRSV (MOI of 0.8)-infected MARC-145 cells, 48 h; quercetin (12.5 μg/mL)- and Rapa (100 nM)-treated MARC-145 cells, p-mTOR/mTOR ratio. (**C**,**D**) PRRSV-infected MARC-145 cells, 48 h; quercetin (12.5 μg/mL)- and MHY1485 (10 μM)-treated MARC-145 cells, p-mTOR/mTOR ratio. (**E**) PRRSV (MOI of 0.8)-infected MARC-145 cells, 48 h; quercetin (12.5 μg/mL)- and MHY1485 (10 μM)-treated MARC-145 cells, ORF7 mRNA expression. *** *p* < 0.001, compared with control. ### *p* < 0.001, compared to the viral group. +++ *p* < 0.001, compared to the Rapa/MHY1485 group.

To investigate the regulatory role of quercetin on the PI3K/Akt/mTOR pathway, we employed the mTOR inhibitor Rapa and the mTOR activator MHY1485 in subsequent experiments. Co-treatment with Rapa and PRRSV further suppressed mTOR phosphorylation, which was reversed by quercetin (Figure 6A,B). In contrast, MHY1485 treatment significantly enhanced mTOR phosphorylation (Figure 6C,D) and reduced PRRSV replication (Figure 6E), indicating a close association between viral replication and PI3K/Akt/mTOR pathway activity. Furthermore, co-treatment with quercetin and MHY1485 further increased mTOR phosphorylation levels and decreased PRRSV copy numbers. Collectively, these results demonstrate that quercetin inhibits PRRSV infection by modulating the PI3K/Akt/mTOR signaling pathway.

## 4. Discussion

PRRSV is an enveloped, positive-strand RNA virus classified under the genus *Porartevirus*, family *Arteriviridae*, and order *Nidovirales*. PRRS remains one of the most economically devastating diseases in the global swine industry, making the development of effective prevention and control strategies of paramount importance. Given that conventional vaccines offer limited protection, the exploration of novel anti-PRRSV therapeutics, including natural compounds such as quercetin, presents a promising avenue for combating this disease [25,26,27].

Autophagy is a critical process for maintaining cellular homeostasis through the recycling and degradation of organelles and proteins [28]. The replication of PRRSV is intricately linked to the autophagic process. Indeed, mounting evidence indicates that the virus can induce autophagy through multiple distinct pathways [29,30]. For instance, PRRSV infection activates Ca^2+^ signaling, leading to Ca^2+^ influx into the cytoplasm from extracellular sources or the endoplasmic reticulum, thereby inducing autophagy. Additionally, PRRSV infection upregulates Rab1a, a GTPase implicated in autophagy, which induces autophagy through the interaction of Rab1a with ULK1. Undoubtedly, PRRSV augments its replication by inducing autophagy. Autophagosomes that form around the viral replication complex offer PRRSV, similar to coronaviruses, a milieu of surrounding DMVs for replication, thereby facilitating PRRSV replication. Concurrently, autophagosomes containing viral components bind to and degrade lysosomes, and excessive autophagy induces apoptosis. Accordingly, PRRSV inhibits the fusion of autophagosomes with lysosomes, thereby inducing incomplete autophagy that can augment its replication [31,32,33,34]. Based on the above findings, we investigated whether quercetin inhibits PRRSV replication via autophagy. Quercetin is defined as an environmentally benign natural compound and a key component of various herbal remedies. It possesses well-documented antiviral properties.

Quercetin, a polyphenolic flavonoid belonging to the flavanone subclass, is found in various edible and medicinal plants [35]. Numerous studies have demonstrated its antioxidant, anti-inflammatory, cardioprotective, antiviral, and antibacterial properties [35,36,37,38,39,40]. Furthermore, quercetin can alter disease progression by regulating autophagy. For example, in lung cancer cells, quercetin reduces p62 protein expression and promotes the fusion of autophagosomes with lysosomes [41]. Additionally, in the human lens epithelial cell line SRA01/04, quercetin regulates the PI3K/Akt/mTOR signaling pathway [42]. In our study, quercetin treatment significantly reduces mRNA and protein expression of Beclin-1 and ULK1, indicating that autophagy is inhibited. This led us to propose the hypothesis that quercetin inhibits the formation of pre-autophagosomes, thereby suppressing the replication of porcine reproductive and respiratory syndrome virus (PRRSV) in vitro. We validated this hypothesis by treating infected cells with Rapa, which enhances PRRSV replication, while quercetin successfully reversed this effect. Moreover, quercetin treatment decreased p62 mRNA and protein levels, further supporting the notion that quercetin may facilitate autophagosome-lysosome fusion to reduce PRRSV replication. This was substantiated by experiments using CQ, an autophagy inhibitor that prevents autophagosome-lysosome fusion, where quercetin continued to suppress viral replication. These findings suggest that quercetin inhibits PRRSV replication by modulating autophagic flux dynamics.

The induction of autophagy by PRRSV is a multi-stage process. In the early phase of infection, the virus triggers complete autophagy, enabling both the initiation of autophagy and the subsequent fusion of autophagosomes with lysosomes. Whereas in the later stages, PRRSV shifts to inducing incomplete autophagy. This latter stage is characterized by the initiation of autophagy that fails to proceed to autophagosome-lysosome fusion, a process believed to support the viral life cycle [43]. In this study, significant lesions were observed 48 hpi, a time point at which IFN-β expression peaked and PRRSV-induced incomplete autophagy was evident. Given the variability in PRRSV strains, further studies are required to investigate the early stages of PRRSV infection and the precise role of quercetin in modulating these events.

To investigate the mechanisms underlying quercetin’s effects, we focused on the PI3K/Akt/mTOR signaling pathway, a key regulator of autophagy and a target of Rapa [44]. Our results revealed that PRRSV infection reduced the phosphorylation levels of PI3K, Akt, and mTOR, while quercetin treatment restored mTOR phosphorylation in infected cells. To further evaluate whether quercetin exerts its antiviral effects through the PI3K/Akt/mTOR pathway, we treated cells with the mTOR inhibitor Rapa or the mTOR activator MHY1485. MHY1485 treatment increased mTOR phosphorylation and decreased PRRSV replication. Co-treatment with quercetin and MHY1485 significantly reduced the phosphorylation level of mTOR and consequently decreased the PRRSV copies, suggesting that quercetin may modulate PRRSV replication via the PI3K/Akt/mTOR pathway**.** The PI3K/Akt/mTOR pathway is a crucial intracellular signaling axis, and further investigation is needed to elucidate the precise molecular mechanisms through which quercetin influences cellular processes within this pathway. Moreover, the PI3K/Akt/mTOR pathway primarily regulates autophagosome formation, necessitating further investigation to elucidate how quercetin enhances autophagosome-lysosome fusion and its subsequent impact on viral replication.

While this study provides detailed mechanistic insights into how quercetin inhibits PRRSV replication through autophagy modulation, we acknowledge its primary reliance on the MARC-145 cell line. Although this is a standard model for initial mechanistic studies of PRRSV, it lacks the species-specific immune milieu of primary porcine macrophages. Therefore, the direct translational relevance of our findings to porcine physiology requires future validation in primary porcine alveolar macrophages (PAMs) or other immunocompetent porcine systems. Such studies would be crucial to confirm the antiviral potency of quercetin and its impact on the PI3K/Akt/mTOR-autophagy axis in the virus’s natural target cells. This represents a critical direction for our subsequent research.

## 5. Conclusions

In conclusion, our study demonstrates that quercetin inhibits PRRSV infection by modulating autophagy, specifically by suppressing autophagosome formation through the PI3K/Akt/mTOR signaling pathway and promoting autophagosome degradation. Given the limitations of conventional vaccine strategies and the absence of specific antiviral treatments for PRRS, quercetin represents a promising natural compound with potential therapeutic value in combating PRRSV infection.

## Figures and Tables

**Table 1:** Sequences of primers (Sangon Biotech, Shanghai, China).

Name	Sequence (5′—3′)
β-actin-F	TGCCTCATGCCATTCTCC
β-actin-R	CTGACCATCTCCTGCTCAA
ORF7-F	CTAAGAGAGGTGGCCTGTCG
ORF7-R	GAGACTCGGGCATACAGCACA
Beclin-1-F	GCTGCCGTTATACTGTTCT
Beclin-1-R	TGCCTCCTGTGTCTTCAA
p62-F	GATAACTGTTCAGGAGGAGAC
p62-R	TCGGATTCTGGCATCTGTA
ATG5-F	ACTTGCTTCACGCTATATCA
ATG5-R	CTCACTAATGTCTTCTTGTCTC
ATG12-F	CCAAGGACTCATTGACTTCAT
ATG12-R	CTCATACAGAGTTCCAACTTCT
IFN-β-F	GAGTGTGGAGACCATCAAGGAAGAC
IFN-β-R	GTTCATGTACTGCTTTGCGTTGGAC

## Data Availability

The original contributions presented in this study are included in the article and Supplementary Material. Further inquiries can be directed to the corresponding author.

## Supplementary Materials

The following supporting information can be downloaded at: https://www.mdpi.com/article/10.3390/v17121637/s1, Figure S1: Quercetin reduces autophagosome. MARC-145 cells were treated with 25 μM CQ or 100 nM Rapa alone or together with 12.5 μg/mL quercetin for 48 h. Autophagosome was detected with MDC staining. Scale bar, 250 μm. Figure S2: Blocking autophagic flux attenuates the inhibitory effect of quercetin on PRRSV replication. MARC-145 cells were treated with 25 μM CQ alone or together with 12.5 μg/mL quercetin for 48 h. (**A**) Autophagosome was detected with MDC staining. Scale bar, 250 μm. (**B**) ORF7 mRNA expression of PRRSV. MARC-145 cells were treated with 25 μM CQ or 100 nM Rapa alone or together with 12.5 μg/mL quercetin for 48 h. Autophagosome was detected with MDC staining. Scale bar, 250 μm.
