# Supplementary figures and images for: Quercetin Regulates Autophagy to Inhibit PRRSV Replication Through the PI3K/Akt/mTOR Signaling Pathway
Source: Viruses. 2025 Dec 17;17(12):1637. doi: 10.3390/v17121637 (PMC12737713; doi:10.3390/v17121637)

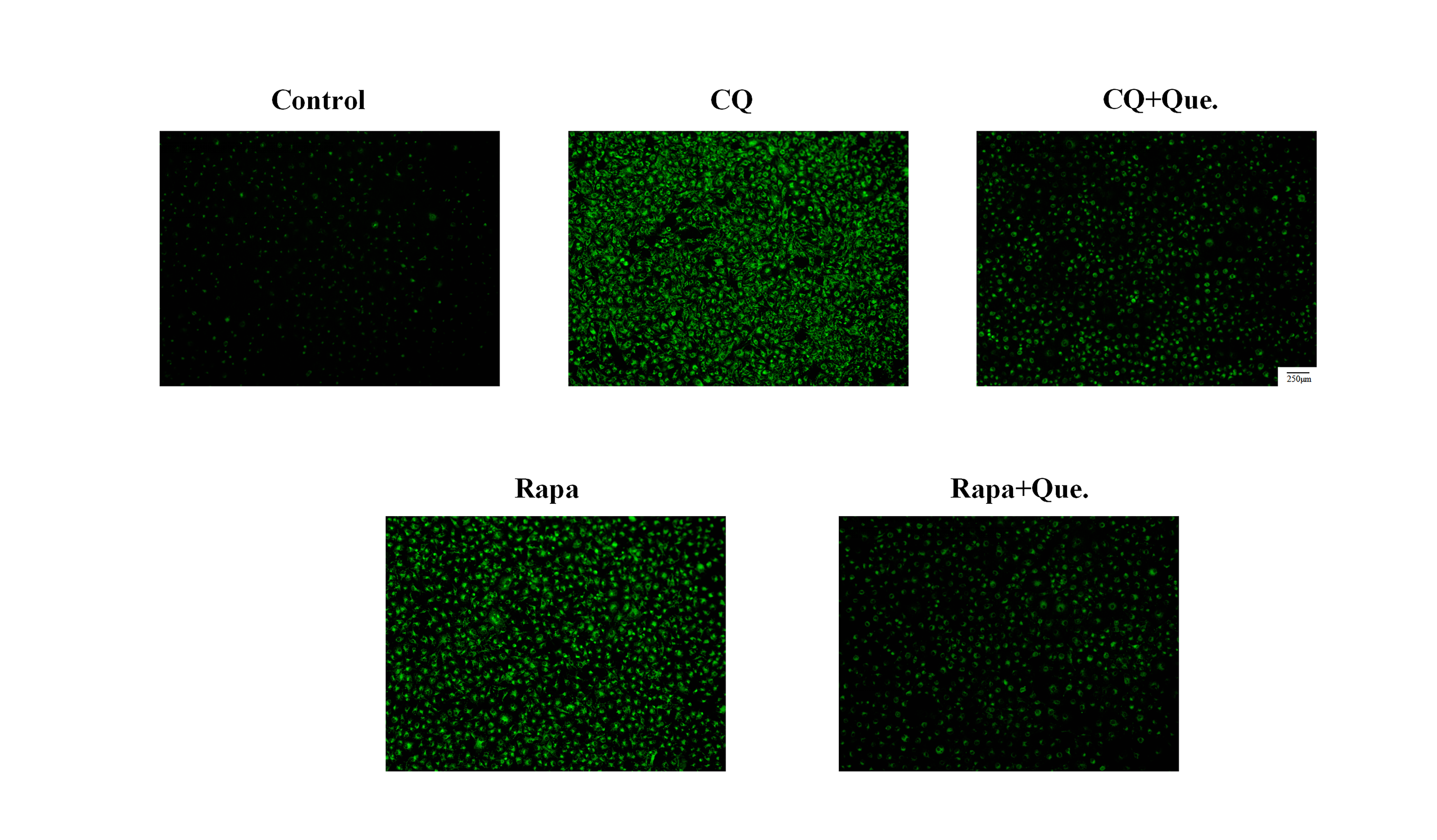

Supplement: Supplementary file 1 [file viruses-17-01637-s001.zip › Figure S1.tif]

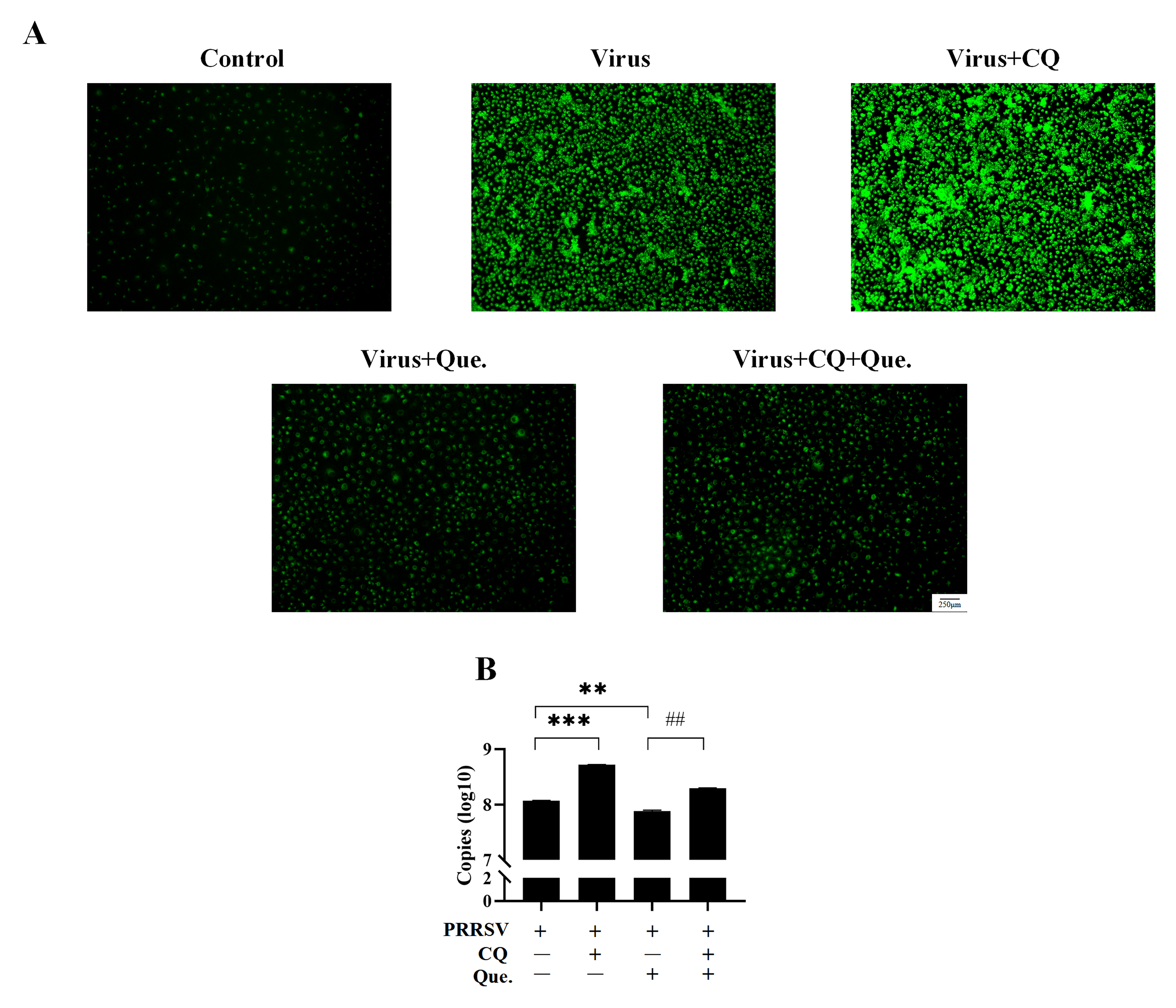

Supplement: Supplementary file 1 [file viruses-17-01637-s001.zip › Figure S2.tif]
